# Using Rational Models to Interpret the Results of Experiments on Accent Adaptation

**DOI:** 10.3389/fpsyg.2021.676271

**Published:** 2021-11-05

**Authors:** Maryann Tan, Xin Xie, T. Florian Jaeger

**Affiliations:** ^1^Centre for Research on Bilingualism, Department of Swedish Language & Multilingualism, Stockholm University, Stockholm, Sweden; ^2^Brain & Cognitive Sciences, University of Rochester, Rochester, NY, United States; ^3^Department of Language Science, University of California, Irvine, Irvine, CA, United States; ^4^Computer Science, University of Rochester, Rochester, NY, United States

**Keywords:** non-native speech, L2 speech, adaptation, distributional learning, ideal observer, computational modeling, rational cognition

## Abstract

Exposure to unfamiliar non-native speech tends to improve comprehension. One hypothesis holds that listeners adapt to non-native-accented speech through distributional learning—by inferring the statistics of the talker's phonetic cues. Models based on this hypothesis provide a good fit to incremental changes after exposure to atypical *native* speech. These models have, however, not previously been applied to non-native accents, which typically differ from native speech in many dimensions. Motivated by a seeming failure to replicate a well-replicated finding from accent adaptation, we use ideal observers to test whether our results can be understood solely based on the statistics of the relevant cue distributions in the native- and non-native-accented speech. The simple computational model we use for this purpose can be used predictively by other researchers working on similar questions. All code and data are shared.

## 1. Introduction

Understanding strongly non-native-accented speech can be challenging: native listeners unfamiliar with a non-native accent tend to process it more slowly and with decreased accuracy (Munro and Derwing, 1995; Witteman et al., 2013). There is now ample evidence that this initial processing disadvantage can decrease with exposure to the accented talker (e.g., Weil, 2001; Bradlow and Bent, 2008; Adank et al., 2009), with some improvements emerging within mere minutes of exposure (Clarke and Garrett, 2004; Xie et al., 2018b). What has remained less well-understood are the mechanisms underlying these changes in speed and accuracy of processing.

Two broad classes of (mutually compatible) hypotheses have emerged. One holds that changes in native listeners' processing of non-native-accented speech arise from a general relaxation of decision criteria for phonological categorization (e.g., “general expansion”, Schmale et al., 2012). The other hypothesis holds that listeners learn talker- or even accent-specific characteristics, including information about specific segmental features and super-segmental properties of the accented speech (e.g., Bradlow and Bent, 2008; Sidaras et al., 2009). This latter hypothesis has received further elaboration: that adaptation to non-native accents is at least in part achieved through distributional learning (Wade et al., 2007; Idemaru and Holt, 2011; Schertz et al., 2015; Kartushina et al., 2016) of the type assumed in exemplar (Pierrehumbert, 2001) or Bayesian theories of speech perception (Kleinschmidt and Jaeger, 2015).

Distributional learning models have been found to provide a good qualitative and quantitative explanation of certain adaptive changes listeners exhibit in response to shifted or otherwise atypical pronunciations by native talkers (Clayards et al., 2008; Bejjanki et al., 2011; Kleinschmidt and Jaeger, 2015, 2016; Theodore and Monto, 2019). This includes changes in categorization boundaries observed in perceptual recalibration (e.g., Norris et al., 2003; Eisner and McQueen, 2005; Kraljic and Samuel, 2006; Drouin et al., 2016) or unsupervised learning paradigms (Clayards et al., 2008; Nixon et al., 2016). However, tests of distributional learning models have almost exclusively been limited to comparatively small deviations from the expected means or variances of two phonological categories along a single phonetic dimension (for examples with two phonetic dimensions, see Hitczenko and Feldman, 2016; Xie et al., 2021a). Whether distributional learning can explain adaptation to the types of more complex deviations from expected pronunciations that are observed in unfamiliar non-native accents is an open question. Specifically, non-native accents differ from the expected native pronunciation along many acoustic and linguistic dimensions, including both supra-segmental and segmental differences. Non-native speech might, for example, realize segmental or supra-segmental categories with means that are shifted relative to native means (Best, 1995; Flege, 1995) and with expanded or reduced variance (Smith et al., 2019; Vaughn et al., 2019; Xie and Jaeger, 2020), including deviation in terms of the relative reliance on different cues to signal the same phonological contrast (Flege et al., 1992; Xie et al., 2017). In short, adaptation to a talker with an unfamiliar non-native accent constitutes a more complex problem than adjustments in response to more limited differences between native talkers, and it is possible that these challenges require a different set of mechanisms (for related discussion see Goslin et al., 2012; Porretta et al., 2017).

We take a hugely simplified step toward addressing this question. Our approach is *post-hoc* and confirmatory (although future work might employ the same approach *pre*dictively prior to data collection). We ask whether a simple model of speech perception (an ideal observer, Clayards et al., 2008; Norris and McQueen, 2008; Kleinschmidt and Jaeger, 2015) can be employed to make informative predictions as to whether exposure to a specific set of non-native-accented speech stimuli is expected to result in detectable adaptation (see also Hitczenko and Feldman, 2016). To demonstrate the potential value of such an approach, we ask whether an ideal observer sheds light on what appeared to be, at first blush, a failure to replicate previous findings from accent adaptation (Eisner et al., 2013; Xie et al., 2017), despite very similar design and procedure.

We emphasize that our goal here is not to convincingly argue that distributional learning is the best explanation for the data at hand. Rather, we aim to demonstrate *how* one can use a simple normative model of speech perception to derive predictions for the perception of, and adaptation to, non-native-accented speech. By comparing the responses of human listeners to the predictions of this computational model, researchers can achieve a clearer sense of which results (null or not) should be treated as surprising (see also Massaro and Friedman, 1990, on the value of normative models for speech perception). While models of speech perception suitable for this purpose now exist (Clayards et al., 2008; Kleinschmidt and Jaeger, 2015), they are rarely employed in the interpretation of experimental results (but see e.g., Lancia and Winter, 2013; Kleinschmidt et al., 2015; Hitczenko and Feldman, 2016; Theodore and Monto, 2019; Xie et al., 2021a). Research in experimental psychology often remains focused on *effects* with less discussion of whether these effects are *predicted by existing theories or models* (see discussion in Jaeger et al., 2019). When models are evoked, it is not uncommon that predictions are attributed to them without verifying that a computational model would actually make those predictions. These practices are arguably particularly problematic when applied to human behavior that is known to be affected by previously experienced input (as is the case for speech perception in general and accent adaptation in particular). Even for theories of speech perception that are conceptually simple, the predictions of these models tend to depend on the statistics of previously experienced speech in non-trivial ways. This is precisely the type of situation in which computational studies can provide a deeper understanding of experimental findings, and prevent misunderstandings of existing theory.

The present report aims to demonstrate how even the *post-hoc* application of computational models to experimental data can aid interpretation. It also holds the potential to reduce the “file drawer” problem (Rosenthal, 1979)—the bias to not publish null results—as well as to pre-empt the “over-interpretation” of null results. As we illustrate below, not every null result is a Type II error; null results can be precisely what a model predicts given the specific stimuli of an experiment. We thus hope this report can serve as a helpful guide for researchers, encouraging experimenters to interpret results relative to theoretical models that are sufficiently specified to make predictions. To this end, this report is accompanied by detailed Supplementary Material written as executable, richly documented, R markdown (Allaire et al., 2021) and compiled into an interactive HTML. These Supplementary Material, along with all data, are shared via the Open Science Framework (https://osf.io/72fkx/). The main text aims to provide a high-level overview of the approach and results.

## 2. The ‘Puzzle’

**Figure 1:**
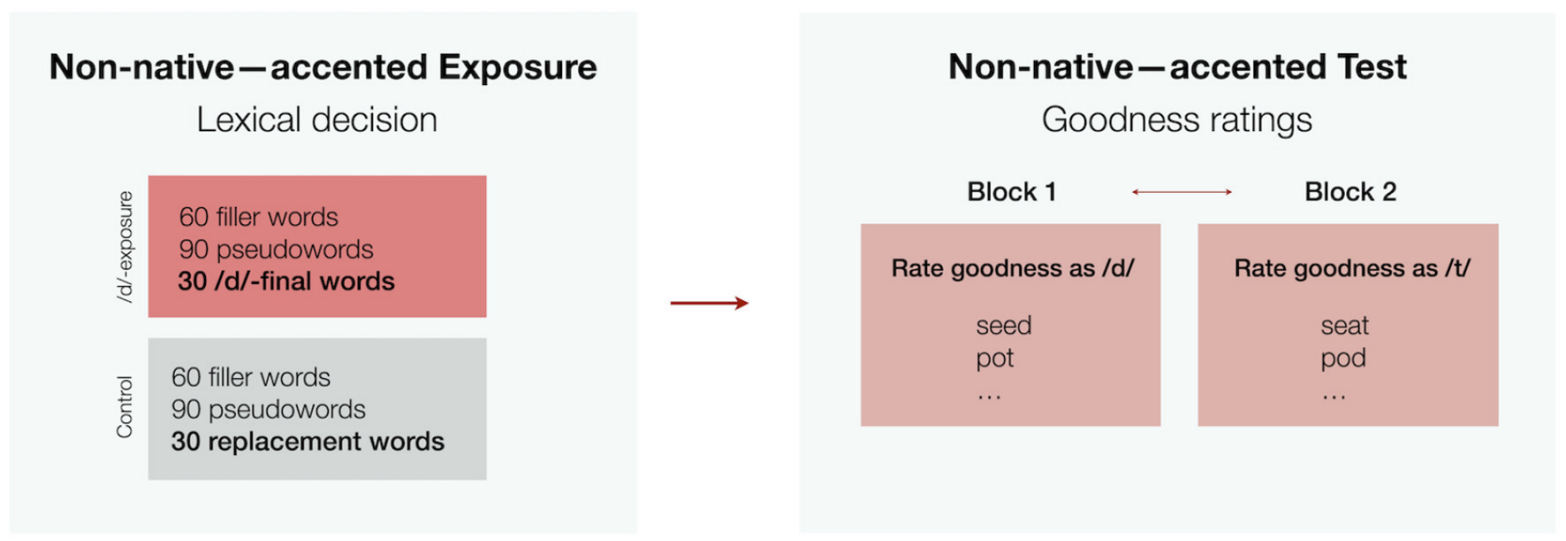
Design of English and Swedish experiment analyzed here. The order of /d/- and /t/-goodness test blocks was counter-balanced across participants.

The two perception experiments we aim to understand share the same exposure-test design and procedure (Figure 1), but differ in the L1-L2 pair investigated. Both experiments investigate adaptation to non-native-accented speech of a single unfamiliar talker (see also Clarke and Garrett, 2004; Eisner et al., 2013, a.o.). Both experiments focus on the realization of the same phonological category—syllable-final stop voicing of /d/, and its contrast to /t/—present in the L2s, but absent in L2 talkers' L1s.

The first experiment exposed native speakers of American English to Mandarin-accented English speech (Xie et al., 2017) while the second exposed native speakers of Swedish to Flemish-accented Swedish. Both Mandarin-accented English and Flemish-accented Swedish are known to differ from native English and Swedish, respectively, in the realization of final stop-voicing (Tan et al., 2019; Xie and Jaeger, 2020). As the Swedish study was designed as a replication of the English study, we describe the English study first.

Unlike English, Mandarin does not have stops in syllable-final position. As would be expected from theories of L2 learning (e.g., Flege, 1995), the realization of final stop-voicing differs between native English and Mandarin-accented English (Flege et al., 1992; Xie and Jaeger, 2020). This was also confirmed specifically for the non-native-accented speech materials used in the experiment (Xie et al., 2017).

Exposure was manipulated between participants. Both groups heard 90 words and 90 pseudowords while conducting a lexical decision task. For the */d/-exposure* group, this included 30 words containing a syllable-final /d/ (e.g., *lemonade*). These exposure words were chosen to not have minimal pair neighbors with syllable-final /t/, allowing lexical guidance on the non-native talker's /d/ productions. Participants in the *control group* heard no words with syllable-final /d/ (for details about the materials, see Supplementary Material). Neither groups heard syllable-final /t/ productions during exposure.

During test, participants in both groups heard the same minimal pair words with syllable-final /d/ or /t/ (e.g., a recording of *seed* or *seat*). Participants had to rate how “good” the word sounded as an instance of /d/ (one block) or /t/ (another block, with the order of blocks counter-balanced across participants). Words within the same minimal pair did not appear in the same block (see Figure 1).

Goodness ratings have been used to analyze listeners' representations of the internal structure of phonological categories (e.g., Samuel, 1982; Volaitis and Miller, 1992; Allen and Miller, 2001), including after exposure to shifted native categories in perceptual recalibration (e.g., Drouin et al., 2016). Xie et al. (2017) found that /d/-exposure led to improved goodness ratings for the non-native-accented /d/- and /t/-final words during test, compared to the control group. We refer to this as the English data. Xie and colleagues replicated the effect of /d/-exposure in three additional experiments using the same recordings and similar exposure-test paradigms but different tasks and participants (Xie and Myers, 2017; Xie et al., 2017, 2018a). The same effect has also been found in experiments with similar designs on syllable-final /d/ in Dutch-accented English, which tends to devoice final stops (Eisner et al., 2013).

In a recent experiment however, we failed to find the effect of /d/-exposure for another L1-L2 pair, Flemish-accented Swedish. Unlike Swedish, Flemish (a dialect of Dutch) devoices voiced stops in syllable-final position (Booij, 1999; Verhoeven, 2005). This type of phonological rule is well-documented to transfer from a talker's first language to their second language and was confirmed in the L2-accented speech materials used in the Swedish experiment (Tan et al., 2019). Like with Dutch- and Mandarin-accented English, we thus expected exposure to Flemish-accented Swedish syllable-final /d/ to affect ratings during test. Both the English and Swedish experiments used lexically-guided exposure with the same task. Both experiments manipulated exposure to the non-native-accented sound (syllable-final /d/) in the same two between-participant conditions, including the same amount of exposure. Both experiments used /d/ and /t/ goodness ratings of /d/-/t/-final minimal pair words during test. Unlike Xie et al. (2017), however, the Swedish data did *not* yield an effect of /d/-exposure on ratings during test. In fact, the effect of exposure went numerically in the opposite direction in the Swedish data.

**Figure 2:**
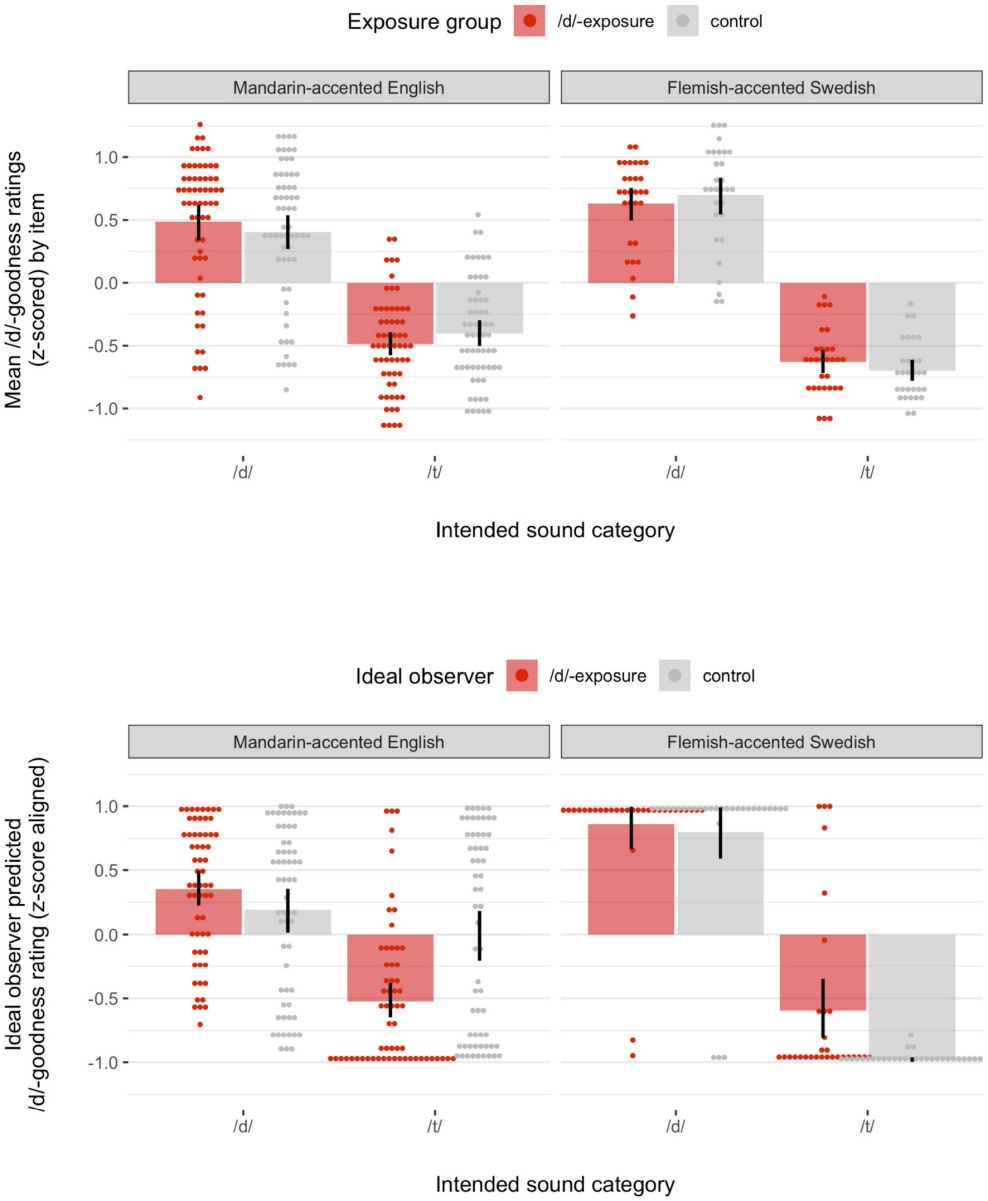
**(Top)** Results of behavioral experiment on native listeners' perception of syllable-final /d/ and /t/ in Mandarin-accented English (left) and Flemish-accented Swedish (right). Points show by-item means of z-scored /d/-goodness ratings (standardized within each participant) for non-native productions of syllable-final /d/ and /t/ during test, depending on the whether participants received exposure to the relevant non-native realization of syllable-final /d/ (/d/-exposure) or not (control). Bars show means and 95% bootstrapped confidence intervals of the by-item means. **(Bottom)** Ideal observer-predicted /d/-goodness ratings described in section 3.2.

Figure 2 (top) shows the rating results from both experiments. Linear mixed-effects regression presented in the Supplementary Material (section 3.2.2) confirmed that the effects of exposure differed significantly between the two experiments (coefficient-based *t*-test, *p* < 0.002): whereas /d/-exposure resulted in significant facilitation for English ($\hat{\beta }=0.04$, *p* < 0.0001), it did not for Swedish—in fact, trending in the opposite direction ($\hat{\beta }=$-0.03, *p* > 0.1).

At first blush, the Swedish data seem to constitute a failure to replicate the English experiment. In particular, since the effect found in the English data has been replicated a number of times, it would be tempting to consider the Swedish result a Type II error (rather than the English result a Type I error). Further, adding to this interpretation, the Swedish experiment collected substantially less data: while the English data consists of 120 ratings each from 48 participants, the Swedish data consist of 60 ratings each from 23 participants—about a fourth of the English data. This would seem to suggest lack of statistical power as a straightforward explanation for the null effect in the Swedish experiment. However, even when the English data was down-sampled to the size and structure of the Swedish data, the difference between the two data sets remained significant 57.6% of the time (out of 1,000 hierarchical bootstrap samples, Supplementary Material, section 3.2.5). For English, the simple effect of /d/-exposure went in the predicted direction 89.6% of the time, reaching significance in 44.4% of all bootstrap samples (vs. 0.6% significant effects in the opposite direction). For Swedish, the simple effect went in the predicted direction 29.2% of the time, and was significant in 7.6% of all samples (vs. 40.2% significant effects in the opposite direction).

Overall, this suggests that power differences alone are unlikely to fully explain the difference between the English and Swedish results. Indeed, the same hierarchical bootstrap analyses found that the Swedish results are very unlikely to result if the English experiment is taken as the “ground truth”: only 12 out of 1000 (1.2%) random resamples of the English experiment resulted in *t*-values as small or smaller than the one observed in the Swedish experiment.

What then caused the difference in results? And do the Swedish data really constitute a Type II error? The Supplementary Material (section 2) discusses a comprehensive list of differences in methodology between the experiments. This comparison revealed that the recordings for two experiments had been obtained in different ways. The Flemish-accented Swedish materials were elicited by first playing a native-accented recording of the word, whereas the Mandarin-accented English materials were elicited without such assistance (Supplementary Material, section 2.2). This raised the possibility that the Flemish-accented Swedish recordings deviated less from native Swedish than the Mandarin-accented English recordings deviated from native English, which would reduce the perceptual benefit of /d/-exposure.

**Figure 3:**
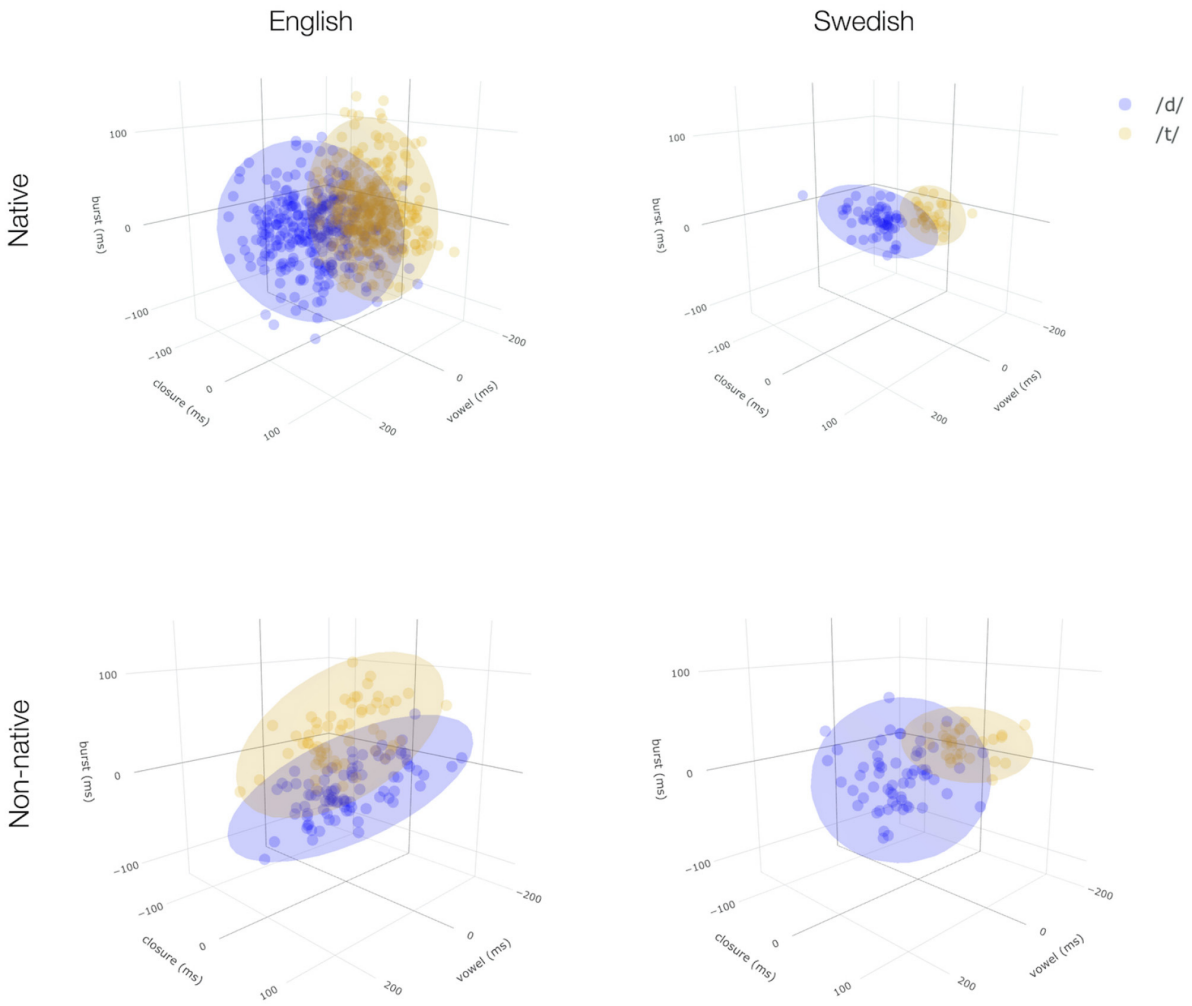
Comparison of native- **(top)** and non-native-accented **(bottom)** syllable-final /d,t/ for both the English (left) and the Swedish (right) data. Productions combine information from multiple databases and are corrected for phonotactic context effects (see Supplementary Material, section 4.3) and are shown in the 3D space defined by three important cues (duration of vowel, closure, burst) to syllable-final voicing. Ellipses contain 95% of the probability mass centered around the mean under the assumption that categories form multivariate Gaussian distributions. To facilitate comparison, axis limits are held constant across panels. See Supplementary Material (section 4.3.2) for interactive visualization, 1D density plots, and 2D pair-wise correlation plots.

An initial comparison of the non-native-accented /d,t/ productions during test to productions of the same test words by a Swedish native speaker (not included in the experiment, but recorded using a similar procedure) lends credence to this hypothesis. Figure 3 shows native- and non-native-accented syllable-final /d,t/ productions of all test items for both English and Swedish. Native productions were obtained from one or more gender-matched speakers similar in age to the non-native speakers employed in the experiments (for details, see Supplementary Material, section 2.2.1). We annotated native- and non-native-accented production for three cues known cross-linguistically to signal syllable-final stop voicing: the duration of the preceding vowel, the duration of the closure interval, and the duration of the burst release (for details on the annotation procedure, see Supplementary Material, section 4.1). The computational studies we present below confirm that these three cues were indeed highly informative about stop voicing in both English and Swedish, though it is possible, if not likely, that listeners employ different (related) or additional cues. Syllable-final stop voicing in Mandarin-accented English is known to differ in the use of these three cues, compared to native-accented English (Xie and Jaeger, 2020), as also clearly visible in the left panels of Figure 3 (replicating Xie et al., 2017). At least at first blush, the Flemish-accented recordings seem to deviate less strongly from the native Swedish productions (right panels) than the Mandarin-accented recordings deviate from native English productions (left panels).

In line with this initial impression, the Flemish-accented Swedish recordings were substantially easier to process for the Swedish participants compared to the Mandarin-accented English recordings for the English participants: lexical decision accuracy during exposure was substantially higher for the Swedish data (Swedish, d-exposure: 96%, control: 97%) than for the English data (/d/-exposure: 78%, control: 74%). This included accuracy on the critical exposure words with syllable-final /d/ (English, /d/-exposure: 78%, SD = 9%; Swedish, /d/-exposure: 94%, SD = 6%; for further detail, see Supplementary Material, section 3.1)^1^.

We thus decided to estimate the predicted consequences for the benefit of /d/-exposure for each experiment given the specific distributional properties of (1) the non-native-accented /d/ in the /d/-exposure group in that experiment, (2) the “typical” native-accented /d/ and /t/ in that language, and (3) the non-native-accented /d,t/-final minimal pair words during test. From this point on—having ruled out a number of alternative explanations for the seemingly diverging results—our approach is confirmatory: our goal is not to rule out alternative mechanisms for accent adaptation but rather to explore how a simple but fully specified computational model of distributional learning can aid data interpretation. This, we hope, may be informative for researchers who find themselves in a situation similar to the one described here: trying to understand (or even predict) the results of an experiment—specifically, the expected results based on the distributional properties of the speech stimuli employed in the experiment.

## 3. Modeling the Effect of Exposure

We approach this question using ideal observers, specifically ideal categorizers, though we note that exemplar models would make similar predictions for the present purpose (for demonstration, see Shi et al., 2010). We use ideal observers because they provide an analytic framework to derive how an ideal/rational listener should respond to input given a certain set of assumptions (for early discussion of the value of this approach, see Massaro and Friedman, 1990). Like exemplar models, ideal observers link distributional patterns in the speech input—which listeners are assumed to have successfully learned, or at least approximated, through exposure (e.g., McClelland and Elman, 1986; Luce and Pisoni, 1998; Norris and McQueen, 2008; for reviews, see MacDonald, 2013; Kuperberg and Jaeger, 2016)—to the categorization decision listeners make during speech perception. Specifically, the posterior probability of recognizing an input as category *c* is a function of both the category's prior probability, *p*(*c*), and the probability of observing the input under the hypothesis that the speaker intended to produce category *c* (the “likelihood”), *p*(*cues*|*c*). These two pieces of information are assumed to be integrated optimally, as described by Bayes' theorem:


(1) $$\begin{array}{r} p\left(c|cues\right)=\frac{p\left(cues|c\right)*p\left(c\right)}{\Sigma_{i}p\left(cues|c_{i}\right)*p\left(c_{i}\right)} \end{array}$$

Just as listeners are assumed to acquire the distributional parameters in Equation (1) from the speech input, researchers can estimate the resulting implicit knowledge of a typical listener from databases of speech production. Of appeal is that this approach makes predictions about *perception* based on only data from *production*, with zero computational degrees of freedom: the likelihood and prior distributions in Equation (1) are fully determined by the production data (unlike in, for example, exemplar models). This makes it noteworthy that ideal observers have been found to provide a good explanation for a variety of phenomena in speech perception and spoken word recognition (e.g., Luce and Pisoni, 1998; Clayards et al., 2008; Norris and McQueen, 2008; Feldman et al., 2009; Bejjanki et al., 2011; Kleinschmidt and Jaeger, 2015; Kronrod et al., 2016).

Here we use ideal observers as a methodological tool to estimate how an idealized participant who has adapted to the phonetic distributions in the input during exposure would respond to the test items. The lack of additional computational degrees of freedom is of particular appeal for this purpose, since fewer degrees of freedom reduce the risk of over-fitting the model to the data. In the same spirit, the models we present in the main text make a number of simplifying assumptions—many of them known to be wrong, but none of them trivially explaining the predictions we derive. These assumptions are summarized in Supplementary Table 1. Here we emphasize only the assumptions that make the models ideal*ized* rather than *ideal* (for the same distinction, see also Qian et al., 2016): rather than model ideal incremental adaptation to the exposure stimuli (Kleinschmidt and Jaeger, 2015), we model listeners that (1) have *completely* adapted by the end of exposure, and (2) do not adapt further during the test phase or at least not much. While (2) is plausible (inputs during test are not lexically labeled since they are minimal pair words; and adaptation seems to proceed most quickly upon initial exposure to talkers, Kraljic and Samuel, 2007), assumption (1) is likely wrong. Indeed, ideal adaptation should weight and integrate the observed input from a talker with prior expectations, so that only partial adaptation is expected after exposure to 30 critical words—partial in the sense that listeners' representations are not a replica of the statistics of the non-native speech, but rather somewhere between the native and non-native speech (Kleinschmidt and Jaeger, 2015).

### 3.1. Methods

We developed four ideal observer models, matching the four combinations of experimental conditions: 2 experiment (Swedish vs. English) X 2 exposure group (/d/-exposure vs. control). Our goal was to approximate the effects of exposure in these four conditions. All models encode listeners' beliefs about /d/ and /t/ as multivariate Gaussian distributions in the 3D space defined by vowel, closure, and burst duration. Category priors, *p*(*c*) in Equation (1), were assumed to be uniform, with each category having a prior probability of 0.5 in all models. This is not meant to entail that syllable final /t/ and /d/ are equally probable in English (they are likely not), but rather that participants expect the two sounds to be equally probable in the context of the experiments (in which they repeatedly observe minimal pair words during test).

To approximate the effect of /d/-exposure, we estimated the mean and covariance of the /d/ category from the 30 non-native-accented recordings of the syllable-final /d/ employed during the experiments' exposure phase. To approximate the effect of control exposure, we estimated the mean and covariance of the /d/ category from recordings of the same 30 exposure words by a gender- and age-matched native speaker. Since by design, neither /d/- nor control exposure contained similarly lexically-labeled instances of syllable-final /t/, we made the simplifying assumption that both idealized listeners would have native /t/ categories. This ignores that listeners might adapt their expectations about /t/ based on exposure to the talker's /d/ or other categories whose realization is correlated with that of the /t/ category (see, e.g., Chodroff and Wilson, 2017). The Supplementary Material describes the databases (section 4.1) and annotation procedure (section 4.2) we employed to estimate the means and covariances of the native /t/ and non-native /t/ and /d/ categories.

While test words formed minimal pairs, holding phonotactic context constant across productions of /d/ and /t/, this was not the case between exposure and test productions. We thus use multiple linear regression to correct cue values for effects of segmental, supra-segmental and talker context (for details, including interactive plots illustrating the consequence of the correction procedure, see Supplementary Material, section 4.3). This approach closely follows the influential C-CuRE model of cue normalization (McMurray and Jongman, 2011), extending it to the contrast between native and non-native speech. C-CuRE has been found to provide a good fit against human categorization responses, including influences of coarticulation due to phonotactic context (Apfelbaum and McMurray, 2015). All ideal observers were fitted to and evaluated on these context-corrected cue values (Supplementary Material, section 4.5).

Both the control and d-exposure ideal observers were then applied to the *non-native-accented* minimal pair words from the test phase of the experiments (Supplementary Material, section 4.6). For each test token, we calculated the ideal observer's posterior probability of /d/ (and /t/), using Bayes theorem. In order to relate the posterior probabilities of /d/ and /t/ to participants' goodness ratings, it is necessary to specify a linking hypothesis. Conveniently, human categorization responses for the same stimuli and the same exposure conditions as analyzed here are available from a separate experiment in Xie et al. Paralleling Xie and colleagues' rating experiment, the categorization experiment found the predicted shift in the /d/-/t/ category boundary following /d/-exposure, compared to control exposure (Xie et al., 2017). This allowed us to investigate the relation between human goodness ratings and proportions of categorization responses, using generalized additive mixed models (GAMMs, Hastie, 2017). These analyses (presented in the Supplementary Material, section 4.6.4) revealed a clearly linear relation between proportion /d/-responses in categorization and /d/-goodness ratings (and, vice versa, for /t/), at least for the type of stimuli analyzed here. For our analyses, we thus assume a simple identity link between the ideal observers' predicted posterior probability of a category and listeners' goodness ratings for that category. For visualizations (e.g., Figure 2, bottom), we facilitate comparison of ideal observers' prediction to human ratings by scaling the ideal observer-predicted posterior probabilities (range = 0–1) to have the same range as human rating responses across the combined English and Swedish data (range = −1 to 1). In those visualizations, we refer to the resulting predictions as posterior ratings. This scaling does not affect correlations between the ideal observers' predictions and human rating responses.

### 3.2. Results: Goodness Ratings Predicted by Ideal Observer

**Figure 4:**
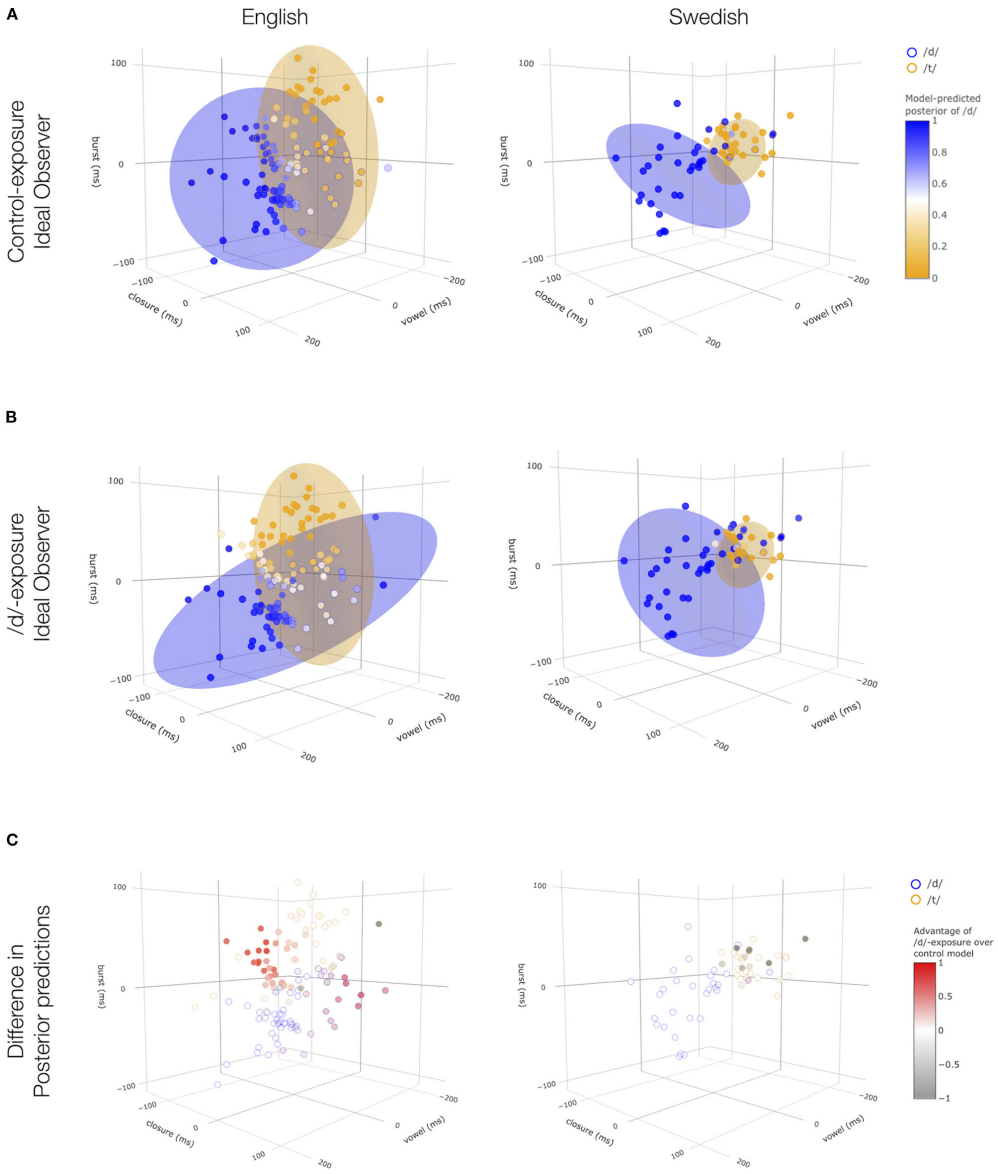
Predicted ratings of control **(A)** and /d/-exposure ideal observers **(B)**, as well as their difference **(C)** shown for all test tokens. These models were constructed from the distributions shown in Supplementary Material (section 4.3.2) so as to simulate a learner who has either been exposed to, and perfectly learned, the statistics of the non-native /d/ (/d/-exposure model) or has not been exposed to non-native /d/ and thus assumes native /d/ statistics (control model). Ellipses visualize the category likelihoods assumed by the respective ideal observers (specifically 95% of the probability density). Across all panels, the outline color of points indicate their intended category. In **(A,B)**, the degree of color match between a point's outline color and fill color indicates a more accurate prediction matching the intended category. In **(C)**, the color fill of points indicate the difference in posterior predictions between the /d/-exposure and control exposure models: redness indicates better performance in the /d/-exposure model (relative to control) and grayness indicates the opposite pattern. See Supplementary Material (section 5.3) for interactive visualization.

Figure 4 (bottom row) shows the results for the control and /d/-exposure ideal observers and both exposure conditions. Paralleling participants' goodness ratings for Mandarin-accented English in Figure 2, posterior ratings were improved under the non-native English model compared to the native English model. And, paralleling participants' goodness ratings for Flemish-accented Swedish, no such improvement of posterior ratings was observed under the non-native Swedish model compared to the native Swedish model. Further analysis presented in the Supplementary Material (section 5.2), confirmed that these results held across randomly sampled subsets of the data (training and test folds).

The ideal observers thus predict effects of exposure condition on goodness ratings that *qualitatively* resemble the results of both the English and the Swedish data. In particular, had we applied the ideal observers to the exposure and test stimuli from both experiments *prior to collecting data*, we would have correctly predicted an effect for the English experiment and a null effect for the Swedish experiment. In this sense then, the Swedish experiment would *not* constitute a Type II error. The quality of fit was also confirmed by trial-level linear mixed-effects regressions reported in the Supplementary Material (section 5.3). These analyses found that the posterior probability of the /d/ category was a significant predictor of listeners' /d/-goodness ratings ($\hat{\beta }=1.22$, *p* < 0.001). This effect remained significant when the experiment (English vs. Swedish), exposure group (/d/-exposure vs. control), and their interaction were included in the analysis ($\hat{\beta }=0.17$, *p* < 0.02; for additional details, see Supplementary Material, section 5.3).

To further elucidate the reason for the differences in the ideal observers' predictions for the two experiments, Figure 4 shows the ideal observers' predictions for each of the items participants heard during test, shown in a 3D cue space. A distributional learning framework predicts failure to observe evidence for adaptation if (a) the non-native exposure stimuli provide misleading information about the non-native stimuli during test or (b) if the distributions of cues in the non-native exposure stimuli do not differ much from native distributions. From the first two rows of Figure 4, it is apparent that the predicted null effect for the Swedish experiment is an example of case b): rather than the /d/-exposure model performing badly on the test items, both the control and the /d/-exposure model perform well on the test items. The reason for this is also obvious: the realization of native and non-native /d/ did not differ much for the Swedish recordings (see also Figure 3). For the English recordings, on the other hand, the cue distributions for the Mandarin-accented /d/ stimuli differed starkly from those of the native-accented /d/ stimuli. Deviating from native pronunciations, the Mandarin-accented talker showed no distinction between /d/ and /t/ in vowel and closure duration but clear separation along the burst dimension (Figure 3, bottom left). This gave listeners in the /d/-exposure group a clear learning advantage over the control exposure group.

## 4. Discussion

Critical reviews of standard practices in the psychological sciences have called out the tendency to dismiss null results as uninformative (Franco et al., 2014). A welcome consequence of this is that it is now easier to publish null results, often as failures to replicate. This reduces the “file drawer” problem (Rosenthal, 1979). The present work can be seen as building on this idea, aiming to understand *why* a null effect is observed. Specifically, the motivation for the present report grew out of an attempt to extend a previously replicated result of accent adaptation to a new L1-L2 pair, Flemish-accented Swedish. Apart from the language, test talker, and lexical materials, this experiment closely followed the design and procedure of previous work, specifically an experiment on Mandarin-accented English (Xie et al., 2017). Beyond the rating results from Xie and colleagues, several other studies with similar design had previously found the predicted effect of /d/-exposure, indexed either by increased auditory priming effects (Eisner et al., 2013; Xie and Myers, 2017; Xie et al., 2017) or improved segment identification (Xie et al., 2017). We thus expected that the experiment on Swedish would find positive evidence of adaptation, yet it seemingly failed to do so. After having ruled out differences in statistical power as a likely cause for the difference in results, we turned to computational models of speech perception to understand whether differences in the statistical properties of the exposure and test stimuli can explain the difference in results.

We found that ideal observers predict both the positive evidence for an effect for Mandarin-accented English in Xie et al. (2017) and the lack thereof in our experiment on Flemish-accented Swedish. This suggests that the original results were not a Type I error, nor are the Swedish results a Type II error. Rather, our ideal observer analyses suggest that the Swedish experiment would not find an effect even if repeated as a large-scale replication, at least as long as the same exposure and test stimuli are used. Indeed, even a much longer exposure phase that repeatedly presents the same non-native /d/ pronunciation as in our experiment on Swedish would not be expected to yield significant changes in participants' goodness ratings. The reason for this is clear from Figure 4: while the Flemish-accented talker differs from native speakers of Swedish in her realization of Swedish syllable-final /d/, these differences are small compared to the non-nativeness observed in the Mandarin-accented speech employed in the experiment on English.

At least qualitatively, ideal observer models provide a good fit against listeners' rating responses. This is noteworthy since the modeling approach employed here does not include *any* degrees of freedom to mediate the effect of input statistics on perception. The only parameters of ideal observers describe the statistics of categories' cue distributions in the speech input. These parameters are thus not fitted to participants' responses during the perception experiment but rather are fixed by data from speech *production*—specifically, speech data that is assumed to have formed listeners' prior expectations based on native speech input and speech data that listeners observe during exposure in the experiment. Based on these speech data, ideal observers make predictions about listeners' *perception* during a subsequent test phase (here goodness ratings). In this sense, ideal observers offer a particularly parsimonious explanation for the differences in results between the two experiments.

The present findings thus are compatible with the hypothesis that adaptation to non-native accents involves similar mechanisms as adaptation to talker-specific differences between native talkers (see also Eisner et al., 2013; Reinisch and Holt, 2014), and that these mechanisms include some form of distributional learning (see also Wade et al., 2007; Xie and Myers, 2017; Xie et al., 2017). Notably, recent work might be seen as calling into question the existence of such shared mechanisms (Zheng and Samuel, 2020). Zheng and Samuel report a failure to find a correlation between individuals' changes after exposure to shifted native speech (perceptual recalibration) and exposure to non-native accented speech (in a paradigm not unlike the present one). However, unlike the present work, the analyses presented by Zheng and Samuel do not assess whether such a correlation would actually be predicted by theories of distributional learning for the particular exposure and test recordings of their study. And, while Zheng and Samuel present power analyses, these analyses are based on arbitrarily selected effect sizes rather than effect sizes expected under theories of distributional learning. This and similar studies are thus an interesting venue for future applications of the modeling approach presented here, allowing researchers to shed light on the informativeness of null findings.

The present study also contributes to efforts to facilitate the theoretical interpretation of perception experiments through computational modeling (e.g., Clayards et al., 2008; Feldman et al., 2009; Toscano and McMurray, 2010; McMurray and Jongman, 2011; Kleinschmidt and Jaeger, 2015; Kronrod et al., 2016; Chodroff and Wilson, 2018). In particular, an emerging body of work has used ideal observers and ideal adaptors to quantify how changes in the distributional statistics of phonetic cues affect listeners' categorization decisions (e.g., Clayards et al., 2008; Kleinschmidt and Jaeger, 2011, 2016; Kleinschmidt et al., 2012, 2015; Theodore and Monto, 2019). When listeners are exposed to speech in which categories' cue distributions deviate from those of typical talkers—e.g., in terms of changes in categories' means or variances—this affects how listeners perceive and categorize subsequent input from the same talker. This manifests in changes in the location (Kleinschmidt and Jaeger, 2011, 2015; Kleinschmidt et al., 2012) or the steepness of listeners' categorization functions (Clayards et al., 2008; Theodore and Monto, 2019) that are well-described by ideal observer and adaptor models. More recent work has begun to go one step further, using exposure-induced changes in categorization behavior from multiple exposure conditions to probe the structure of listeners' prior expectations about cross-talker variability (Kleinschmidt and Jaeger, 2016; Kleinschmidt, 2020).

Previous work in speech perception has employed ideal observers mostly for 2AFC or n-AFC tasks (ideal categorizers, e.g., Clayards et al., 2008; Hitczenko and Feldman, 2016; Xie et al., 2021a). However, with suitable link functions, ideal observers can be applied to other types of tasks and dependent variables. Ideal observers have, for example, been used to model perceptual discrimination (Feldman et al., 2009; Kronrod et al., 2016) and sentence transcription (Xie et al., 2021b, Supplementary Material). Here, we have extended them to model category goodness ratings from 7-point Likert scales (see Supplementary Material, section 4.6.4).

In the present study, we used one case study to demonstrate how computational modeling aids the interpretation of experimental results that run counter to expectations. But computational models can provide substantial gain even when the result of experiments seemingly conform to expectations. A case in point that is directly relevant to the present study comes from recent work by Hitczenko and Feldman (2016). Like the present work, Hitczenko and Feldman employed computational models *post-hoc* to inform the theoretical interpretation of a previously reported finding from an experiment on adaptation to a synthesized accent (Maye et al., 2008). Maye and colleagues exposed listeners to synthesized American English in which all front vowels were simulated to have undergone phonological lowering (e.g., [i] became [ı] and [ı] became [ε], etc.). Listeners subsequently completed a lexical decision task of previously unheard words by the same synthesized voice with front vowels either lowered or raised. Based on the specific pattern of results, Maye and colleagues concluded that listeners adapted to the synthesized accent by shifting the means of their category representations, rather than merely becoming more accepting of *any* type of input. This finding and its interpretation has been influential, with almost 300 citations since 2008. Hitczenko and Feldman (2016) revisit these results, comparing them to the predictions of different types of ideal distributional learners (ideal adaptors, an extension to the simpler ideal observers employed here Kleinschmidt and Jaeger, 2015). Based on these computational comparisons, Hitczenko and Feldman conclude that shifted category representations are *not* the only way, or even the best, way to explain the specific changes in listeners' perception after exposure to the synthesized accent.

### 4.1. Limitations and Future Directions

These studies and the present work serve as examples of how computational models can inform the theoretical interpretation of empirical findings. One strength of the computational approach is that it compels deeper introspection about the assumptions that are necessary to derive predictions from a theory, and to make those assumptions explicit. We refer the reader to Table 4.2 in the Supplementary Material, which aims to list all assumptions we made in the present study. In the remainder, we discuss some of these assumptions, their limitations, and how future work might go about relaxing and revising them.

First, we made simplifying assumptions about what sources of noise contribute to listeners' estimates of the relevant cue distributions. Acoustic noise in the environment and neural noise in listeners' perceptual systems distort the speech signal produced by talkers beyond whatever variability results from noise during the planning and execution of speech articulation. By estimating distributions from speech recordings, our ideal observers ignore whatever acoustic noise our participants experienced beyond those in the recordings, as well as any noise within listeners' perceptual systems^2^. This might explain why the responses predicted by the ideal observers are more categorical than the actual responses made by human listeners: adding perceptual noise to our ideal observers would increase the variance of cue distributions, leading to more shallow categorization functions, and thus less categorical predicted rating responses. Previous work has demonstrated that noise effects can be quantitatively estimated from separate perceptual data and integrated into ideal observers (Feldman et al., 2009; Kronrod et al., 2016). It would be informative to see whether the inclusion of perceptual noise improves the fit between the ideal observers' predictions and human perceptual decisions.

Second, we applied normalization procedures on the acoustic cues to correct for phonotactic context effects. We made the simple assumption that—for native listeners, whose perception we were aiming to model—such correction is based on previous experience with native speech, rather than being shaped by the exposure to non-native speech in the experiment. That is, neither the control, nor the /d/-exposure model assumed learning of non-native phonotactic regularities. On the one hand, this would seem to be in the spirit of C-CuRE and related normalization approaches (Lobanov, 1971; Nearey, 1978; McMurray and Jongman, 2011). For example, C-CuRE computes acoustic cues relative to expectations about the mean of cues in a particular phonotactic or talker context. Critically, the C-CuRE model presented in McMurray and Jongman (2011) assumes that these adjustments are made independent of each other—i.e., this normalization procedure corrects for talker-specific differences in cue distributions and for phonotactics, but not for talker-specific phonotactics. On the other hand, there is evidence that non-native speech deviates from native speech in not only the overall realization of categories, but also in how specific phonotactic contexts affect pronunciation (as found in, e.g., Flege and Wang, 1989; Lahiri and Marslen-Wilson, 1991; Xie and Jaeger, 2020). Whether listeners in the accent adaptation experiments learn these non-native phonotactics in addition to changes in category-to-cue distributions is an open question. Future work could therefore compare models like ours without learning talker- or accent-specific phonotactic patterns against models that also learn this information.

Third, we constructed the /d/-exposure and the control models directly from the input statistics in each accent (non-native vs. native). These models assumed complete learning whereby listeners are assumed to have fully converged toward exposure statistics. In reality, rational listeners are expected to be guided by prior beliefs based on their native experience. While such priors facilitate adaptation to talker-specific statistics that meet prior expectations (Kleinschmidt and Jaeger, 2015), the same priors slow-down and constrain learning of unexpected non-native statistics (Kleinschmidt and Jaeger, 2016; Kleinschmidt, 2020). Learners are thus not expected to fully converge against the statistics experienced during exposure. Future work might consider the same type of incremental Bayesian belief updating applied in previous work on the perception of native speech (Kleinschmidt and Jaeger, 2011; Theodore and Monto, 2019) or synthesized speech (Hitczenko and Feldman, 2016) to investigate adaptation to the perception of non-native speech.

Fourth, and related to the third point, we adopted an assumption commonly made in research on accent adaptation—that participants were unfamiliar with the non-native accents in the experiments. This assumption is almost always questionable. We followed previous work (Reinisch and Holt, 2014), and asked participants to guess the native language of the talker. Based on this measure, participants in either experiment did not seem to be familiar with the accent prior to the experiment. However, explicit identification of accents is likely an unreliable measure of participants' previous experience with an accent (McCullough, 2015; McKenzie, 2015; Gnevsheva, 2018). It is thus possible that some of the results we discussed here are due to participants prior familiarity with the L2 accent in the experiment. Indeed, additional analyses reported in the Supplementary Material (section 5.4) found that participants in the English experiment might have had prior familiarity with Mandarin-accented English or similar L2 accents. The effects observed by Xie et al. (2017) thus do not necessarily reflect the same adaptation as listeners that are completely unfamiliar with Mandarin-accented English or similar L2 accents: on the one hand, prior familiarity might lead to faster adaptation; on the other hand, prior familiarity likely would reduce the difference between the two exposure conditions, since it means that both groups of participants have exposure to Mandarin-accented /d/. As pointed out by a reviewer, it is further possible that Swedish listeners were more familiar with Flemish-accented Swedish (or similar accents) than L1 English listeners are familiar with Mandarin-accented English (or similar accents). This would provide an alternative explanation for the null results in the Swedish experiment. The additional analyses in the Supplementary Material (section 5.4) did not, however, reveal support for this possibility. If anything, these analyses argued against this possibility though we note that the lack of a significant exposure effect makes it difficult to rule it out entirely (see discussion in the Supplementary Material).

Beyond the aforementioned specifics of the models, there are limitations to the specific way in which the present study employed ideal observers: our approach has been both *post-hoc* and confirmatory. With regard to the latter, future work could follow in the footsteps of Hitczenko and Feldman (2016), and compare the ideal observers developed here against alternative hypotheses. For example, instead of distributional learning, the effects of different exposure on listeners' rating responses during test might reflect changes in response biases (Clarke-Davidson et al., 2008) or a general relaxation of response criteria (Hitczenko and Feldman, 2016). Similarly, future work might employ the same methods we have used here *post-hoc*, but do so *predictively* prior to conducting the experiment. As we have illustrated here, the distributional statistics of the specific input—and more specifically the way in which such statistics differ between native and non-native speech—can be linked to predicted changes in subsequent perception. Future work could, for example, use ideal observer-predicted categorization or rating responses in power analyses to inform experimental designs prior to the experiment (for similar approaches in other domains, see Jaeger et al., 2019; Bicknell et al., in revision^3^).

Finally, it is important to recall that the reliability and generalizability of the results presented here is limited by two types of data sparsity. First, we used phonetically annotated databases to estimate the implicit distributional knowledge that listeners are hypothesized to have learned from previous speech input. Even for well-studied languages like English, these databases tend to be small. For Swedish, we had access to only one talker. While efforts were taken to record a ‘typical’ talker of Swedish, with the hope that the phonetic distributions of this talker would be representative of what native listeners might have come to expect through a lifetime of exposure, the results reported here might change once a larger database with more Swedish talkers is considered. In short, the fact the we obtained a decent fit against human performance for both experiments does *not* show that the amount of data we used to develop the ideal observers was sufficient. Additional analyses presented in the Supplementary Material (section 5.2) address this question. By subsetting both the training and test data for the ideal observers into multiple separate folds, we find that the qualitative match between model predictions and human ratings seems to be surprisingly robust even for the small data sets we had access to. We do, however, also find that the results are considerably more robust for English (trained on 6 native talkers) than for Swedish (trained on 1 native talker). Overall, the results of these additional analyses suggests (1) that 15 training tokens per category and 15 test tokens per category *can* be sufficient for the type of analysis conducted here, but that (2) having access to data from multiple talkers is important for the estimation of listeners' prior (in this case native) knowledge. The second way in which data sparsity limits the conclusions we can draw from the present study is likely more severe. It is also shared with the majority of work on talker-specific accent adaptation: both experiments analyzed here employed a single non-native accented talker. There is now evidence that the results of such experiments can depend on the specific talker (for evidence and discussion, see Xie et al., 2021b). Moving forward, the same models employed here for talker-specific adaptation can be used to understand adaptive changes in listeners' perception and categorization following exposure to multiple talkers, or listeners' ability to generalize previously experienced input to unfamiliar talkers (for discussion and model development, see Kleinschmidt and Jaeger, 2015, Part II.

## Data Availability Statement

The datasets presented in this study can be found in online repositories. The names of the repository/repositories and accession number(s) can be found at: https://osf.io/72fkx/.

## Ethics Statement

The studies involving human participants were reviewed and approved by University of Connecticut Institutional Review Board (for the English version). The patients/participants provided their written informed consent to participate in this study.

## Author Contributions

XX and MT conducted the behavioral experiments in English and Swedish, respectively. TJ and XX led in the statistical analyses. MT contributed to the statistical analyses. All authors contributed to the writing of the manuscript.

## Funding

This study was supported by a travel grant from the Knut & Alice Wallenberg Foundation (2018) awarded to MT.

## Conflict of Interest

The authors declare that the research was conducted in the absence of any commercial or financial relationships that could be construed as a potential conflict of interest. The reviewer MK declared a shared affiliation with one of the authors, XX to the handling editor at the time of the review.

## Publisher's Note

All claims expressed in this article are solely those of the authors and do not necessarily represent those of their affiliated organizations, or those of the publisher, the editors and the reviewers. Any product that may be evaluated in this article, or claim that may be made by its manufacturer, is not guaranteed or endorsed by the publisher.

## Footnotes

^1^The difference in exposure accuracy could also be explained if the Swedish participants were more familiar with accents that involve syllable-final devoicing than the American participants. For example, exposure to German-accented Swedish is common in Stockholm (as our Swedish colleagues were eager to point out). Post-experiment surveys found that none of the Swedish participants was able to guess the L1 of the non-native accent, and only one (4.3%) of the participants guessed another L1 that leads to syllable-final devoicing (German). It is possible, however, that participants nevertheless had subconscious familiarity with syllable-final devoicing. This would explain the lack of an effect of exposure. It would not, however, explain the differences in the degree of accentedness in the *productions*, shown in Figure 3. We also note that analyses presented in the Supplementary Material (section 5.4) suggest that, if anything, prior familiarity with the L2 accent was higher amongst the participants in the English experiment, compared to participants in the Swedish experiment.

^2^At the same time, our ideal observers' estimates of all relevant cue distributions are likely perturbed by measurement errors due to the annotation procedure we used.

^3^Bicknell, K., Bushong, W., Tanenhaus, M. K., and Jaeger, T. F. (in revision). Listeners can maintain and rationally update uncertainty about prior words.
